# Letters from Mars. Soft law under scrutiny of ethics during crisis. Responsibility and accountability: Serge Morard

**DOI:** 10.1093/eurpub/ckac131.223

**Published:** 2022-10-25

**Authors:** S Morard, S Guinoiseau

**Affiliations:** 1 Centre Hospitalier de Crépy-en-Valois, Crépy-en-Valois, France; 2 Centre Hospitalier Layon Aubance, Martigné Briand, France

## Abstract

Like most of western countries France has been severely hit by the first wave of covid during Spring 2020. France managed to get through it thanks to a confinement, full dedication of health and << first line >> workers and of course the excellency of its bureaucracy. France is now famous for its digitalized self-made one-hour-walk certificate. It is however less-known for its MARSes, acronym for << message d'alerte rapide sanitaire >> or quick sanitary warning messages that got usually sent to hospitals and nursing homes... on Friday nights or weekends. Some lawyers saluted the method as an innovative and modern process that adapted quickly the response to the evolution of the situation shifting from hard rules written in the due process of law to an era of properly crafted regulations. Professionnals were less enthusiastic regarding this matter. It was less an issue of professional curtesy expected form a central state towards civil servants than one related to the content of the messages. Quality varied depending on the news. Some messages imposed very clear and strict rules such as: << no visit under any circumstances in nursing homes >> or later when tests were available, << should any cluster appear, all residents must be tested. >> However, all messages ended the same and ritual way: << Provisions included in this message must be performed and adapted under direction supervision and adapted to the context of the institution >>. Interestingly enough the way the text was written rose questions: If the rule is clear, why should it be adapted? The rule enters in conflict with other preexisting rules. Which one should apply? Classical legal and political theory are of great help sorting formal issues. Hume’s non ethical cognitivism and Kant’s categorical imperative were used redesigning the working frame. Since accountability was for direction to bear, so was it for it to draw fair and acceptable solutions in due respect of democratic principles.

**Key messages:**

• Limit cases prove that rules are unsufficient in decision making.

• Balanced decisions require collegiality to overstep limited rationality.

